# Supplementary material for: The changing health impact of vaccines in the COVID-19 pandemic: A modeling study
Source: Cell Rep. 2023 Mar 15;42(4):112308. doi: 10.1016/j.celrep.2023.112308 (PMC10015104; doi:10.1016/j.celrep.2023.112308)
Supplement: Document S1. Figures S1 and S2 and Tables S1 and S2 [file mmc1.pdf]

Cell Reports, Volume *42*

Supplemental information

The changing health impact of vaccines

in the COVID-19 pandemic: A modeling study

Jamie A. Cohen, Robyn M. Stuart, Jasmina Panovska-Griffiths, Edinah Mudimu, Romesh G. Abeysuriya, Cliff C. Kerr, Michael Famulare, and Daniel J. Klein

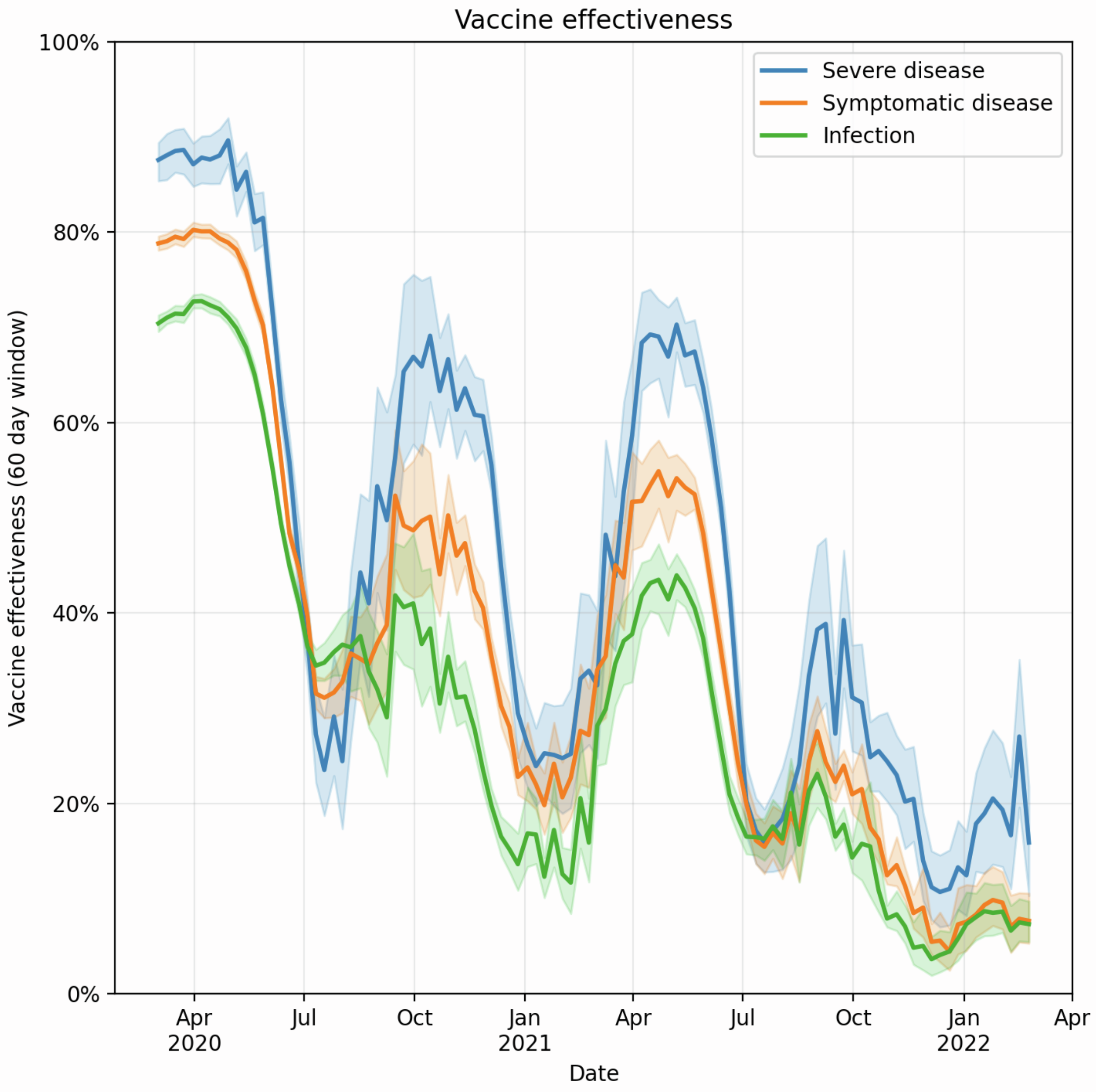

Fig. S1. Vaccine effectiveness against infection, symptomatic COVID and severe disease.

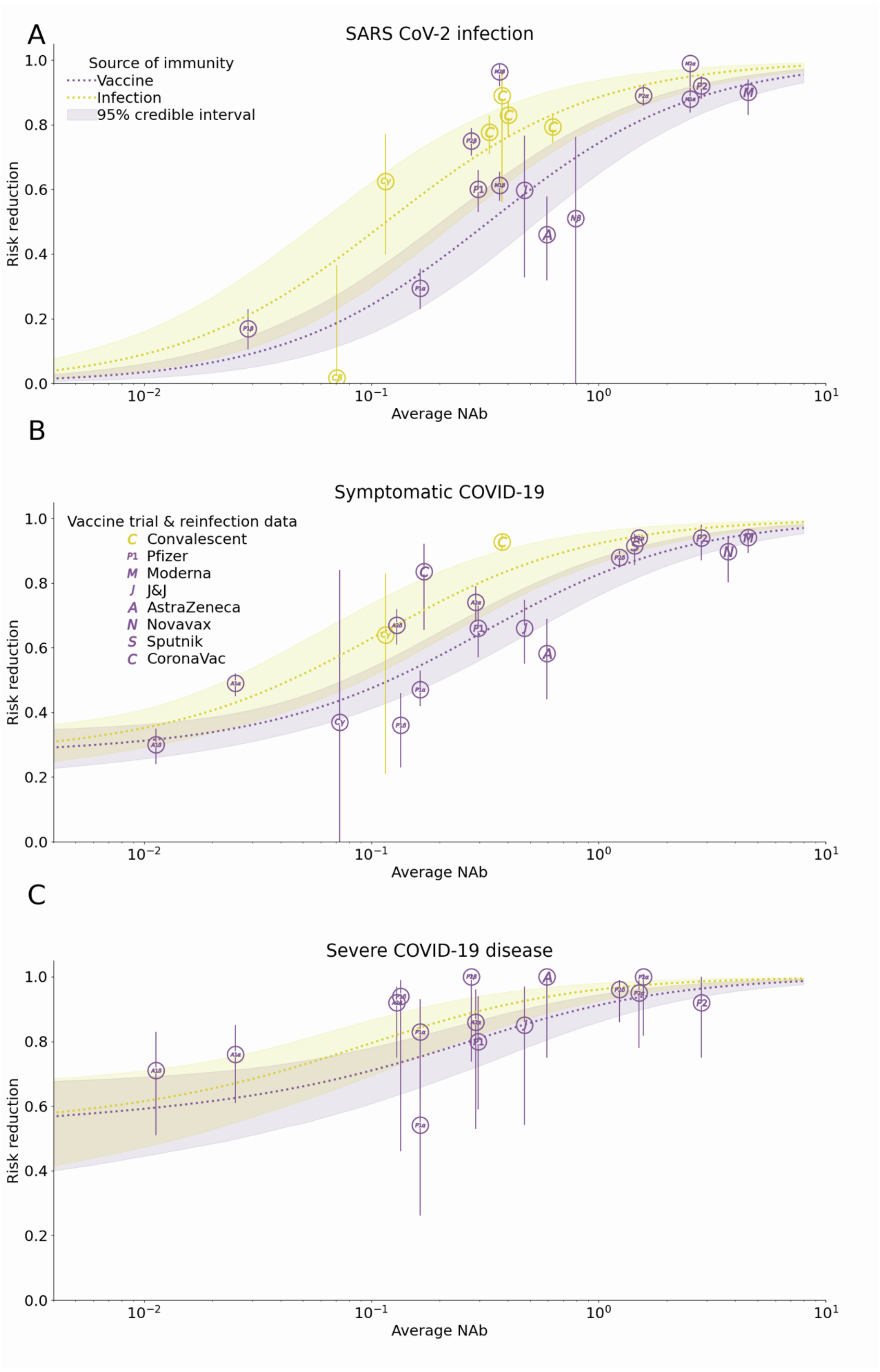

A
SARS CoV-2 infection
Source of immunity
Vaccine
Infection
95% credible interval
Risk reduction
Average NAb
B
Symptomatic COVID-19
Vaccine trial & reinfection data
C Convalescent
P1 Pfizer
M Moderna
J J&J
A AstraZeneca
N Novavax
S Sputnik
C CoronaVac
Risk reduction
Average NAb
C
Severe COVID-19 disease
Risk reduction
Average NAb

Fig. S2. Conditional protection against symptomatic and severe disease given a breakthrough infection and breakthrough symptoms, respectively.

	Mean	Standard Deviation
αinf	1.08	0.226
αnatinfdiff	1.0	0.403
βinf	0.967	0.055
αsymplinf	-0.739	0.187
βsymplinf	0.038	0.0317
αsevlsymp	-0.0143	0.311
β sevlsymp	0.0799	0.078
α_γ	0.639	0.177

Table S1. Fitted parameter values based upon HMC algorithm with 30,000 iterations across 5 chains.

Variant	Infectivity	Severity	WT cross-immunity	Beta cross-immunity	Delta cross-immunity	Pfizer cross-immunity
Beta	1	3.6	6.6%	100%	8.6%	10%
Delta	2.2	3.2	37.4%	8.6%	100%	33%
Omicron	3.0	0.8	5%	4%	4%	2.5%

Table S2. Variant characteristics. Infectivity and severity values refer to the change in per-contact transmission rate and per-infection probability of developing severe disease compared to wild-type. Cross-immunity refers to how much prior neutralizing antibodies are retained to effectively protect against each variant.
