# Supplementary material for: The changing health impact of vaccines in the COVID-19 pandemic: A modeling study
Source: Cell Rep. 2023 Mar 15;42(4):112308. doi: 10.1016/j.celrep.2023.112308 (PMC10015104; doi:10.1016/j.celrep.2023.112308)
Supplement: Document S2. Article plus supplemental information [file mmc3.pdf]

Cell Reports

The changing health impact of vaccines in the COVID-19 pandemic: A modeling study

Graphical abstract

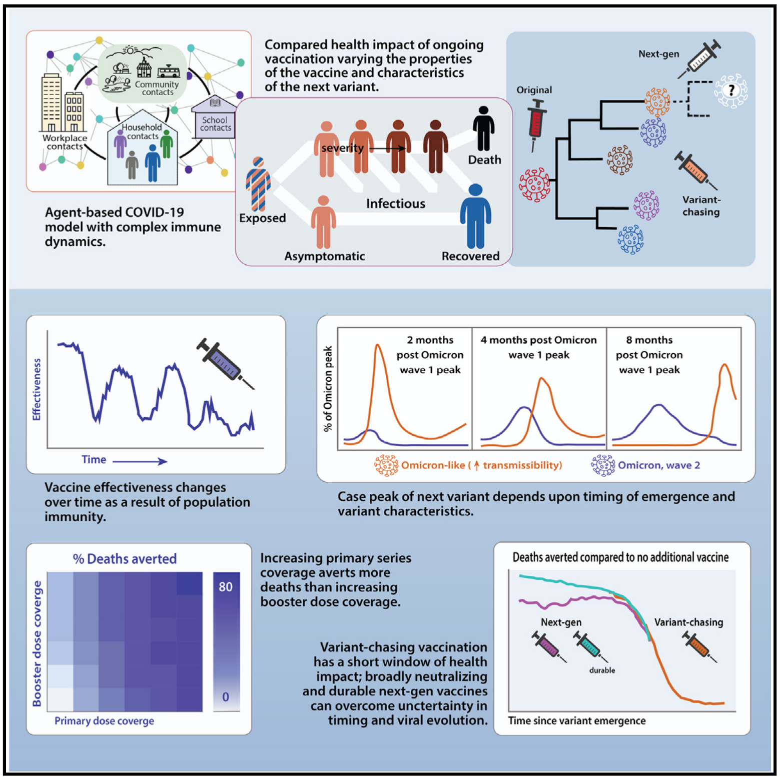

Highlights

- Value of ongoing vaccination depends upon the characteristics of future variants of concern
- Variant-chasing vaccination has a short window of health impact
- Next-generation vaccines can overcome uncertainty in pace and degree of viral evolution

Authors

Jamie A. Cohen, Robyn M. Stuart, Jasmina Panovska-Griffiths, ..., Cliff C. Kerr, Michael Famulare, Daniel J. Klein

Correspondence

jamie.cohen@gatesfoundation.org

In brief

Cohen et al. conduct a model-based analysis of the health impact of COVID-19 vaccination in the context of viral evolution. Cohen et al. find that variant-chasing vaccines have a narrow window of dominating pre-existing vaccines and that next-generation vaccines might be able to overcome uncertainty in pace and degree of viral evolution.

Cohen et al., 2023, Cell Reports *42*, 112308
April 25, 2023 © 2023 The Author(s).
https://doi.org/10.1016/j.celrep.2023.112308

CellPress

Cell Reports

CellPress
OPEN ACCESS

Article

The changing health impact of vaccines in the COVID-19 pandemic: A modeling study

Jamie A. Cohen,[1,7,*] Robyn M. Stuart,[2] Jasmina Panovska-Griffiths,[3,4] Edinah Mudimu,[5] Romesh G. Abeysuriya,[6] Cliff C. Kerr,[1] Michael Famulare,[1] and Daniel J. Klein[1]
[1]Institute for Disease Modeling, Global Health Division, Bill & Melinda Gates Foundation, Seattle, WA, USA
[2]Gender Equality Division (contractor), Bill Melinda Gates Foundation, Seattle, WA, USA
[3]The Big Data Institute and the Pandemic Sciences Institute, University of Oxford, Oxford, UK
[4]The Queen's College, University of Oxford, Oxford, UK
[5]University of South Africa, Pretoria, South Africa
[6]Burnet Institute, Melbourne, VIC, Australia
[7]Lead contact
*Correspondence: jamie.cohen@gatesfoundation.org
https://doi.org/10.1016/j.celrep.2023.112308

SUMMARY

Much of the world's population had already been infected with COVID-19 by the time the Omicron variant emerged at the end of 2021, but the scale of the Omicron wave was larger than any that had come before or has happened since, and it left a global imprinting of immunity that changed the COVID-19 landscape. In this study, we simulate a South African population and demonstrate how population-level vaccine effectiveness and efficiency changed over the course of the first 2 years of the pandemic. We then introduce three hypothetical variants and evaluate the impact of vaccines with different properties. We find that variant-chasing vaccines have a narrow window of dominating pre-existing vaccines but that a variant-chasing vaccine strategy may have global utility, depending on the rate of spread from setting to setting. Next-generation vaccines might be able to overcome uncertainty in pace and degree of viral evolution.

INTRODUCTION

In 2020, the world achieved the fastest vaccine development timeline in history, resulting in multiple highly efficacious vaccines against severe acute respiratory syndrome coronavirus 2 (SARS-CoV-2) available to the public in 11 months. Clinical trials showed that these vaccines dramatically reduce the risk of SARS-CoV-2 infection, mild symptoms, and severe COVID-19.[1–4] Despite the remarkable success of the vaccines, their value is ever changing, as a larger share of the global population has some immunity from either infection and/or vaccination and as the virus evolves to evade immunity and spread more efficiently.[5]

Most COVID-19 vaccines are designed to target the spike protein and stimulate production of neutralizing antibodies that fight infection and CD4+ and CD8+ T cells, which stop disease progression.[6] Neutralizing antibodies wane, contributing to falling vaccine effectiveness against infection over time and against immune evading variants.[7] At the same time, memory B and T cells are relied upon to stimulate an even broader response when the body is exposed to a new virus. Severe disease protection has been more durable because the disease course for COVID-19 occurs on timescales more amenable to the response time of memory B and T cells and less impacted by viral evolution.[8–12]

Even as the vaccines were first being rolled out in late 2020, new variants were already beginning to emerge, and the efficacy of the vaccines was reliant on them providing sufficient cross-protection against these new variants. After the Omicron BA.1 variant was first detected in late 2021, it quickly established global dominance and led to a wave of new infections that peaked almost five times higher than previous waves. By March 2022, the WHO reported that an estimated 90% of the global population had antibodies against the COVID-19 virus through vaccination and/or infection.[13] In the immediate aftermath of a wave of infections of this scale, the impact of vaccination is likely to be reduced, but it is unclear by how much and for how long this immunity will last before the value of vaccination begins to increase again.

Throughout 2022, global infections were primarily comprised of sublineages of Omicron, including BA.2, BA.3, BA.4, BA.5, and descendant lineages. These were all sufficiently immune evading to cause fresh waves of infections, but with variation in the relative scale compared with the original Omicron BA.1 wave. The combination of waning immunity and the continued emergence of immune-evading variants prompted many jurisdictions across the world to deliver booster vaccines throughout 2022 and beyond, which provide increased protection against infection and broaden the immune response. Bivalent vaccines encoding both wild-type and Omicron mRNAs have been developed and shown to induce greater neutralizing antibody responses against Omicron than monovalent vaccines.[14] The attraction of the

This is an open access article under the CC BY license (http://creativecommons.org/licenses/by/4.0/).

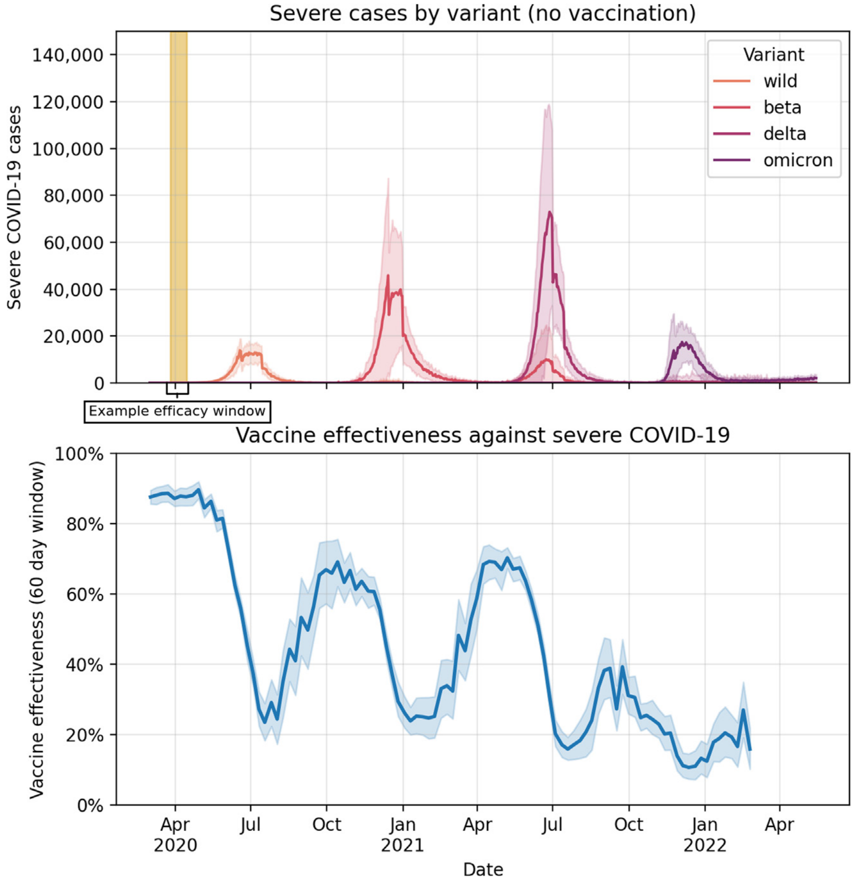

Figure 1. Population-level vaccine effectiveness as a function of time

The top panel shows new severe cases by variant in the absence of vaccination. The bottom panel shows vaccine effectiveness against severe disease over time, which is calculated for the 60 days following vaccine completion (i.e., after second dose). The golden rectangle represents an example vaccine effectiveness window. Simulations are for a South African population of 60 million people.

mRNA platform lies in its potential for developing variant-targeted vaccines to maximize vaccine effectiveness against immune-evading variants.[15]

In this study, we explore the changing value of vaccines in a landscape of dynamic immunity and rapidly evolving variants of concern. We use Covasim, an established agent-based model enhanced with detailed intra-host dynamics, to perform our analyses.[16] We base our study on a South African population, including population size, demographics, and previous infection- and vaccination-derived immunity, selecting South Africa both because it was among the first countries in which Omicron was identified and because previous work that our group had done in modeling COVID-19 in South Africa meant that we had a good departure point for creating a model to explore these questions. We evaluate the population-level effectiveness (quantified as the reduction in the risk of severe disease for vaccinated individuals compared with unvaccinated individuals) and efficiency (quantified by doses per death averted) of vaccines over time and as a function of population immunity from infection and then generalize these results to consider the marginal impact of expanded vaccine coverage and ongoing boosters given uncertain and unpredictable future variants. As the marginal impact of vaccines falls, we evaluate the broadening of our prevention tools to reduce the impact of COVID-19.

RESULTS

Vaccine effectiveness over time

We begin by evaluating population-level vaccine effectiveness as a function of time and incremental to prior immunity. We find that vaccine effectiveness peaks in the month prior to each emerging wave and declines rapidly thereafter. Vaccine effectiveness is highest at the beginning of the pandemic and declines over time as the percentage of the population with immunity from prior infection increases. Within our simulated population representative of South Africa, vaccine effectiveness decreases from close to 90% in the earliest stages of the pandemic to less than 20% in January 2022 in the wake of the Omicron BA.1 wave (Figure 1B). Vaccine effectiveness increases more in advance of a more virulent strain, as seen in the level of protection in advance of Delta, despite 50% prior exposure, compared with a less virulent strain, as seen in the level of protection against Omicron (see Figure 1).

The efficiency of vaccination is also a function of time, with efficiency decreasing over time at an increasing rate. At its peak efficiency, fewer than 100 doses would have been required to avert a single death (or put another way, under 50 people needed to be fully vaccinated). By the end of January 2022, following the Omicron wave, nearly 4,000 doses (or 2,000 people vaccinated) would be required to avert a single death. In comparison, an analysis of childhood and adolescent routine and non-routine immunizations for 10 pathogens estimated an average of 208 fully vaccinated people per death averted over 2000–2019.[17]

The effect of new variants

In the absence of any additional vaccination or new variants, we estimate that a second wave of Omicron would emerge and peak approximately 6 months after the peak of the first wave (see top row of Figure 2). This second wave would be considerably muted given the level of population immunity from the first wave of Omicron. We next explore the epidemic dynamics for each of the variants and timelines considered. Once we consider the emergence of a new variant, we find that the impact of a new variant is more sensitive to the characteristics of the variant (i.e., antigenic

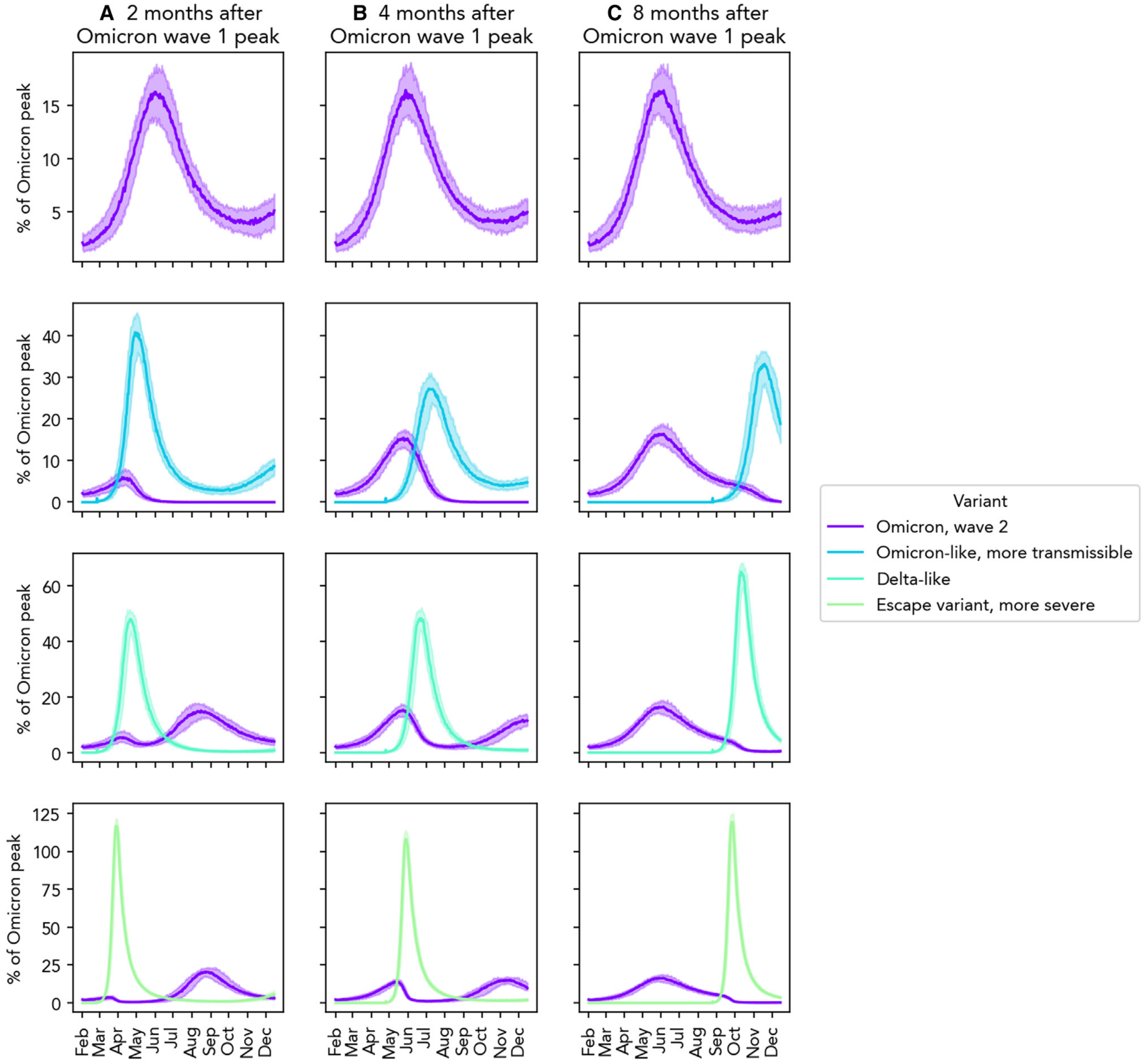

Figure 2. New infections as a percentage of Omicron peak for each of the new variants and variant introduction dates considered
All plots are in the absence of any additional vaccination ("status quo"), other non-pharmaceutical interventions, or behavior change. Columns A–C indicate timing of new variant introduction relative to first wave of Omicron. Rows indicate the characteristics of the new variant. Note that the top row represents a second wave of Omicron without any competition from a new variant.

distance from sources of prior immunity and transmissibility) than the timeline of introduction (i.e., months after prior Omicron wave). We find a dose response with the level of overall immune escape of the new variant and the size of its impact in terms of new infections (see Figure 2).

A variant that emerges from Omicron (i.e., antigenically closest to the most recent variant) but with increased fitness advantage through intrinsic transmissibility would compete directly with a second wave of Omicron. These analyses were conducted in February of 2022, prior to the emergence of the multiple sublineages of Omicron that proceeded to dominate transmission over the ensuing 12 months, but the results shown in the middle panel of the second row of Figure 2 are closest to what ensued in reality, with South Africa experiencing a new wave of infections in May 2022 that peaked at 25% of the value of the peak of Omicron BA.1 infections. The date of introduction is highly influential in the resulting epidemic dynamics. If this variant emerges within 4 months after Omicron BA.1 (Figure 2A), it might dampen and even outcompete the re-emergence of Omicron. If the variant emerges later, the second wave of Omicron would push out the growth of the new variant (Figures 2B and 2C).

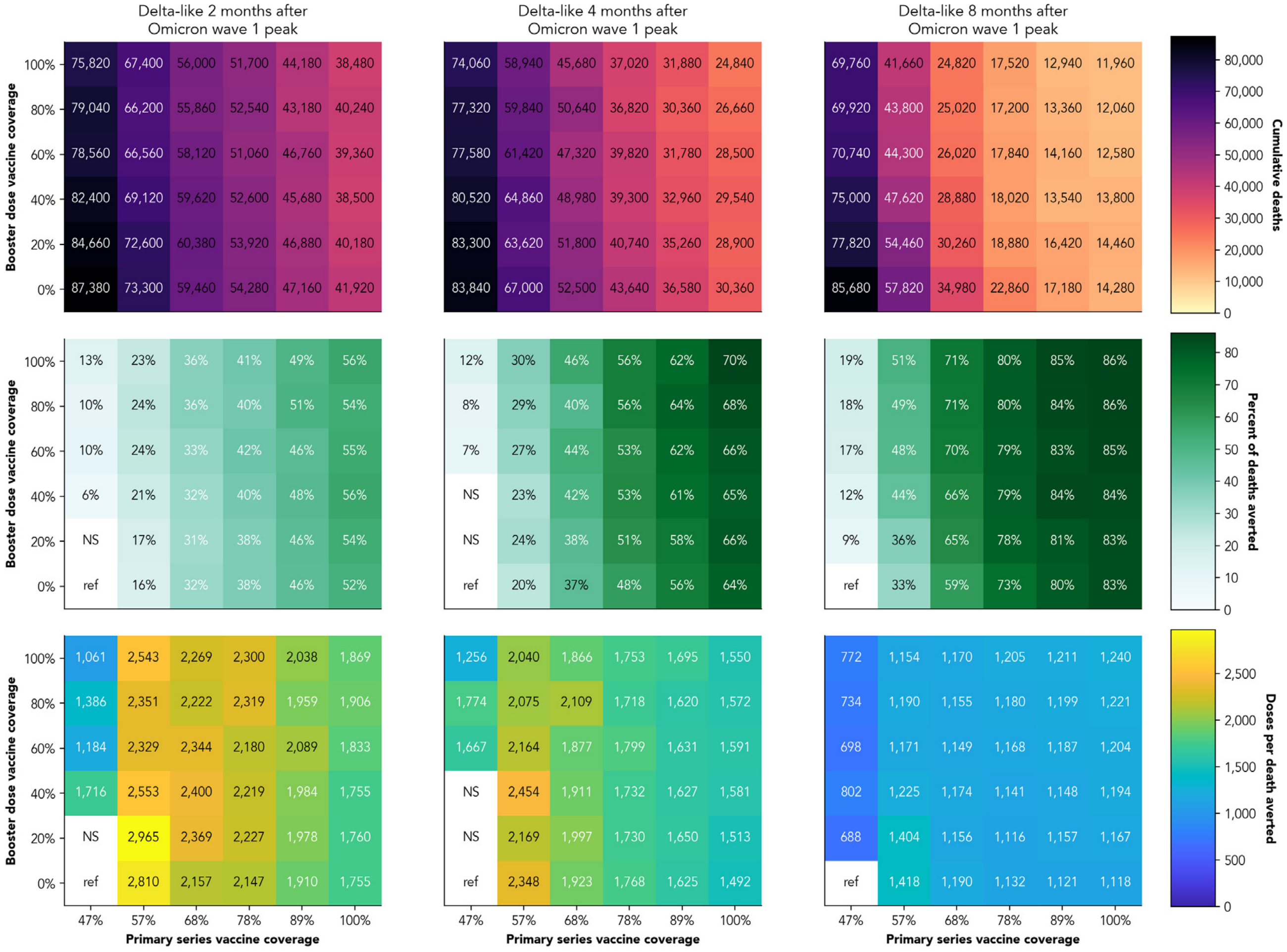

Figure 3. Vaccine impact and efficiency across primary series and booster dose coverage levels for a new Delta-like variant that emerges 2, 4, or 8 months after the peak of the Omicron wave

Booster dose coverage refers to the percentage of already vaccinated individuals who receive a booster dose. The x axis coverage values were chosen to reflect the overall vaccine coverage in the population. Before any additional scale-up, vaccine coverage was at 47%, so each value represents increasing coverage by 20%, 40%, 60%, and 80%. NS (not shown) represents scenarios with zero difference.

When considering the specific variants that we constructed for these analyses, we find that the variant most antigenically similar to Omicron BA.1 would result in a wave of cases 20%–40% of the BA.1 wave peak; the variant most similar to historical variants (labeled in Figure 2 as Delta-like) would lead to a wave of cases 50%–70% of the BA.1 wave peak; and the variant furthest antigenically from all prior immunity would lead to a wave up to 120% of the height of the BA.1 peak. We note that these values are illustrative and intrinsically linked to the properties of the hypothetical variants that we constructed but that the general pattern that emerges from these analyses is that variants that evade prior sources of immunity result in higher peaks in cases and are less sensitive to timing of introduction.

Trade-off between primary series and booster dose coverage

In this section, we quantify the dynamic trade-off between increasing primary series (first and second-dose) and booster dose coverage of vaccination. We focus first on a relatively well-matched vaccine and variant (proxied here by a Delta-like variant, which we assume evades 50% of vaccine neutralizing antibodies [NAbs]), to narrow in on the trade-off between primary series and booster dose coverage, before considering the ongoing impact of antigenic drift. Results indicate that increasing primary series coverage (i.e., decreasing the number of unvaccinated individuals in the population) would have a greater marginal and absolute health impact than increasing booster dose coverage among already vaccinated individuals (see Figure 3). The relative impact varies depending upon the date of the introduction of the new variant, but generally speaking, increasing primary dose coverage would achieve 95% of the total possible benefit of increasing coverage among all individuals. As coverage increases, the numbers of doses per death averted also increase.

We next consider how the trade-off between primary series and booster dose coverage changes based upon how well matched the vaccine is antigenically to the emerging variant of

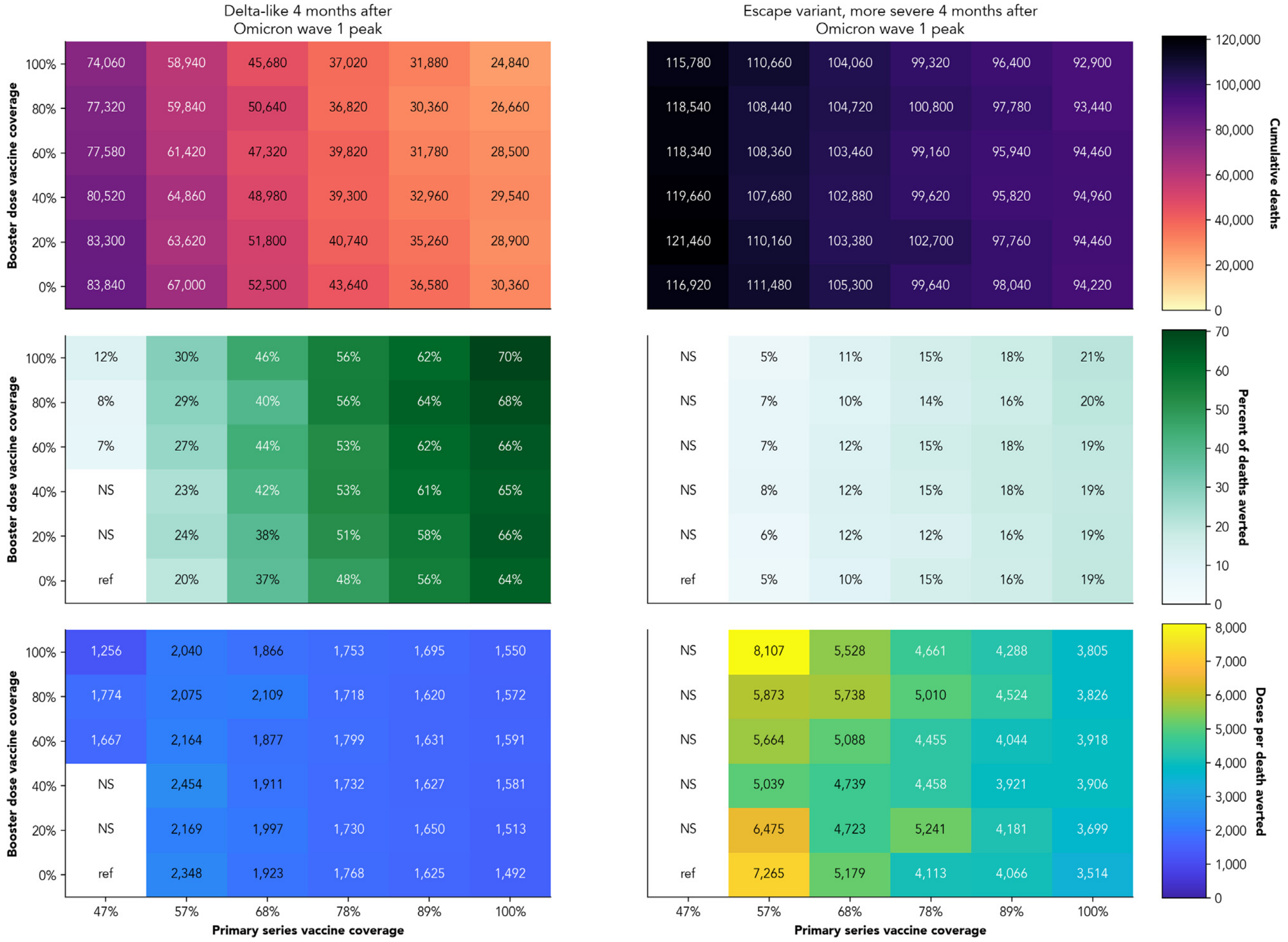

Figure 4. Vaccine impact and efficiency across primary series and booster dose coverage levels for variants of different antigenic distance from vaccination introduced 4 months following Omicron averaged across 50 replicate simulations

Booster dose coverage refers to the percentage of already vaccinated individuals who receive a booster dose. The x axis coverage values were chosen to reflect the overall vaccine coverage in the population. Before any additional scaleup, vaccine coverage was at 47%, so each value represents increasing coverage by 20%, 40%, 60%, and 80%. NS represents scenarios with zero difference.

concern, now fixing the date of the new variant emergence and varying its level of immune escape (comparing a variant that evades 50% of vaccine and prior ancestral cluster NAbs with one that evades 98% of all prior NAbs, choosing these two extremities to bound the likely range of results). Results suggest that vaccination will avert a smaller percentage of deaths (maximally 21% compared with 70% for a more antigenically similar variant; see the top right corners of the second row of Figure 4) and will be less efficient overall (nearly 2-fold more doses per death averted; see the top right corners of the third row of Figure 4) against a variant that evades nearly all vaccine immunity. However, there is more value in increasing primary series vaccine coverage in terms of percentage of deaths averted: starting from a baseline of 47% primary series coverage, a 20% increase in primary series coverage could avert an additional 20% of deaths assuming a Delta-like variant, whereas even the maximally optimistic scenario of 100% booster coverage would not avert this many deaths (see columns 1–2 of the middle left panel of Figure 4). We note, however, that even averting a small percentage of deaths may still correspond to a large absolute number (e.g., achieving full booster coverage would avert almost 10,000 deaths, as shown in the first column of the top left panel of Figure 4). Furthermore, given the smaller number of people needed to vaccinate in order to achieve higher booster coverage levels, and given the fact we are modeling a single-dose booster vs. a two-dose primary regimen, scaling up booster coverage may be comparatively more efficient than scaling up primary series. We find, for example, that full booster coverage implies approximately 1,200 doses per death averted, while scaling up to full primary series coverage implies almost 1,500 doses per death averted (bottom left panel of Figure 4, column 1 vs. row 1).

Variant-chasing and next-generation vaccines

The results from the previous sections suggest that the effectiveness and efficiency of vaccination is highly dependent on the timing and antigenic distance between a renewed vaccine effort

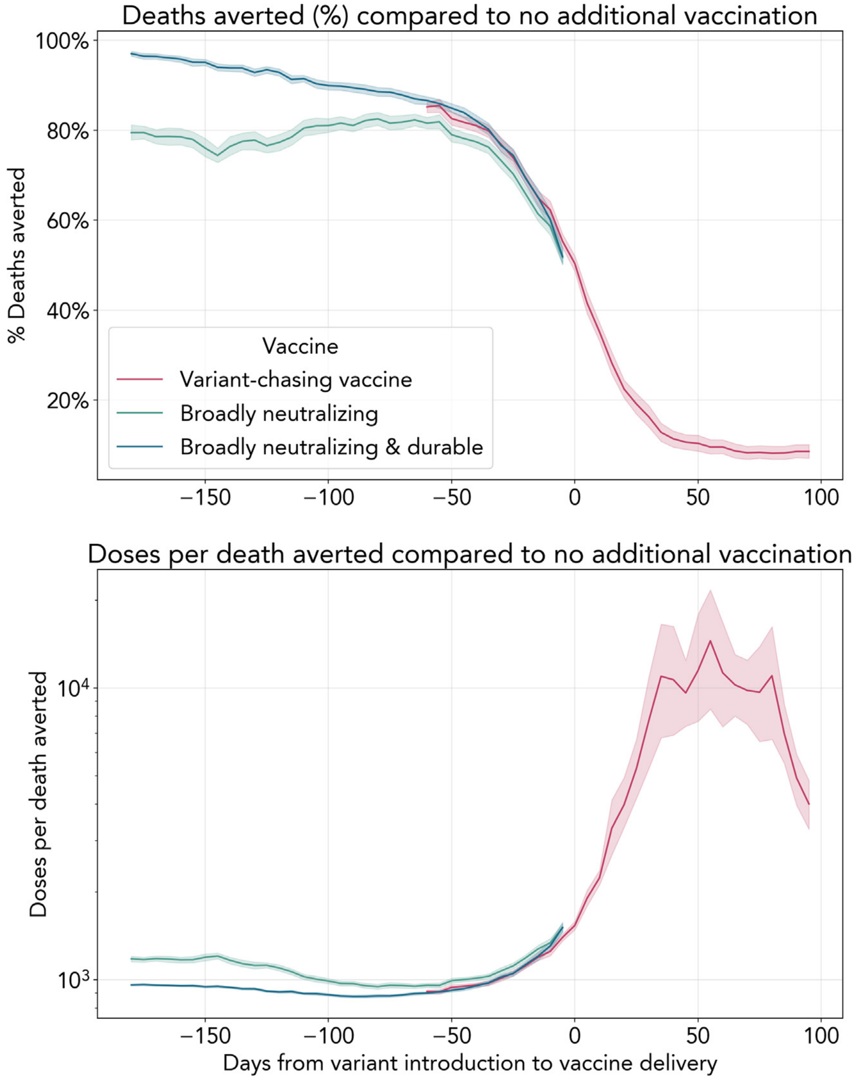

Figure 5. Health impact and efficiency of variant-chasing and next-generation vaccines associated with a variant that emerges 8 months following Omicron, evades 98% of all prior immunity, and is 3.5× more severe than the ancestral virus

Panels show percentage of deaths averted and doses per death averted based on when a variant-specific or next-generation vaccine is rolled out, relative to the emergence of a new variant. In these panels, line color indicates which vaccine is being rolled out. Negative days imply that the vaccine is rolled out before the variant is introduced, which might occur in a global variant-chasing strategy, where sequencing in the emergent country can inform vaccine delivery in other countries before the variant spreads. Variant-chasing vaccine scenarios assume it takes 30 days to reach the target coverage levels once vaccine begins rollout. Next-generation vaccine scenarios assume it takes 90 days to achieve target coverage levels.

and the emerging variant. In light of these findings, we next explored whether a variant-chasing vaccine strategy is effective. We defined a variant-chasing vaccine as the rapid adaptation of our existing vaccines to variant-ready vaccines and boosters that target the specific variant.

The results in Figure 5 illustrate that a variant-chasing vaccine strategy has rapidly declining effectiveness and efficiency the longer it takes to deploy a variant-specific vaccine following variant introduction. Here, we concentrate on the specific example of a variant that emerges 8 months after Omicron BA.1 and evades 98% of all prior immunity against infection. In terms of infections, this effectively equates to introducing the new variant into a naive population, although there is still considerable immunity against severe disease. The date of introduction of a new variant into a population is impossible to know, but Omicron's first emergence was estimated to occur a month before it was first detected.[18,19] We find that if a variant-chasing vaccine could be introduced 1–3 months after introduction (i.e., as soon as detected), it would avert around 10%–20% of deaths.

This model has not accounted for geospatial effects that might enable shorter timelines to be possible in some geographies that learn about the emergence of a new variant from other parts of the world. For example, it is estimated that Omicron emerged in early October 2021,[18] was identified on November 9, 2021, and was reported to the WHO two weeks later, after identification of its numerous amino acid changes.[19] Two days later, it was classified as a variant of concern. By this point, it had spread and approached fixation in South Africa, and so a variant-chasing vaccine in this setting would have had minimal impact. However, it might have had a larger impact in other settings that had not yet seen the importation and/or fixation of Omicron, such as in the United States, where Omicron was estimated to arrive in late November; in Pakistan, India, Brazil, Bangladesh, and Mexico in late December; or in Indonesia and Russia in the middle of January.[20] To capture the global utility of variant chasing, we extended the days from variant introduction to vaccine delivery up to 2 months prior to introduction. One can then reinterpret this figure as representing the possible lag time between variant introduction in the emerging country and vaccine rollout in a second country. The results suggest that there is approximately a 5-week window where developing and deploying a variant-specific vaccine could avert over 50% of deaths, a 2.5-fold higher impact than vaccinating the same share of the population with the current vaccine. After this time, the benefit begins to fall the longer it takes a vaccine to be deployed. A variant-chasing strategy would avert more deaths than current vaccines at the same coverage levels until 20 days after the introduction of the new variant into a population.

Given the falling effectiveness of vaccines over time in common populations with complex immune histories and in the face of immune-evading variants, and the challenge of a variant-chasing

vaccine strategy, we consider the impact of next-generation vaccines that provide broader and more durable (i.e., more slowly waning) immune protection. A vaccine that elicits antibodies that can bind to many diverse strains could protect against highly mutable pathogens, such as SARS-CoV-2.[21] Additionally, a vaccine that provides more durable protection is important in the absence of a seasonal and predictable virus.

Results show that delivering a broadly neutralizing and durable vaccine could avert over 80% of deaths caused by a highly immune-evading, virulent variant if delivered over a month before a new variant emerges (see Figure 5). However, the impact of breadth alone would fall if the timing of delivery was poorly matched to the timing of the next variant. A durable and broadly neutralizing vaccine would overcome the importance of perfectly timing the vaccine and variant chasing, and would provide over 5-fold higher impact than vaccinating the same share of the population with the current vaccine against a highly immune-evading variant.

DISCUSSION

In this work, we used an agent-based model, Covasim, extended to model intra-host immunity, to explore the changing impact of vaccines over time and in the context of emerging immune-evading variants. Our first set of results showed the extent to which vaccine effectiveness varied over the first 2 years of the pandemic. We showed that the impact of vaccination is highest if delivered in the month prior to an emerging epidemic wave and declines rapidly in its aftermath and that population vaccine efficiency, in terms of doses per death averted, falls over time. Within our simulated population, vaccine effectiveness fell to its lowest point (<20%) in the immediate wake of the Omicron wave in December 2021, translating to a 40-fold increase in the number of doses needed to avert a death in early 2022 (post-Omicron) compared with July 2020.

Our second set of results suggest that increasing vaccination coverage among the unvaccinated has a greater marginal and absolute health impact than increasing booster dose coverage among already vaccinated individuals but that the impact and efficiency of vaccination is highly dependent on the timing and antigenic distance between the vaccine start and the emerging variant. Even though our analyses were carried out on a hypothetical population representative of South Africa, we believe this finding will be broadly generalizable given the consistent protective benefit of vaccination against severe disease across different countries and contexts. However, these results may not be robust if our core assumption around durable and evolution-invariant protection against severe disease is fundamentally altered. Furthermore, these results are based on assumptions about individual behavior, government policies, and societal norms (such as masking, lockdowns, and physical distancing) that are likely to differ significantly between regions. For these reasons, while we would expect qualitative similarity between regions in terms of vaccine efficacy, there would almost certainly be large quantitative differences, as we have seen in COVID-19 health outcomes and vaccination rates to date.

When we consider the implementation of variant-chasing vaccines, we find that while this strategy may have limited impact in the countries in which a variant emerges, it may be a globally effective solution, especially if paired with temporary non-pharmaceutical interventions and improved surveillance that could cut the detection time and possibly slow the spread through local restrictions. Alternatively, next generation vaccines that are broadly neutralizing and durable might be necessary as we move into the next phase of COVID-19 endemicity.

SARS-CoV-2 has proven to be a highly adaptable virus, with multiple new variants having emerged throughout 2021–2022 and beyond. Going forward, it is nearly certain that evolution will continue and that new variants will emerge.[22–24] However, assuming we reach a more stable equilibrium where variants emerge in a seasonal and/or predictable pattern, we may find that some of the tools explored here provide options to prevent global spread and burden.

Our results have revealed that a vaccine-only approach has a health impact ceiling. Like the flu, a realistic pathway to improving breadth may be vaccinating with variant-specific vaccines to generate broader immunity to now-relevant strains, even if these vaccines are late to stop first waves. Modeling of this approach can be a useful tool for assessing individual- and population-level impact and cost effectiveness as these vaccines are developed. Additionally, oral antiviral pills may provide a supplemental stopgap measure for reducing disease severity upon the emergence of a new variant. This strategy is more agnostic to timing than vaccination because it can be delivered within 5 days of symptom onset to achieve the full benefit.[25] However, while they may be subject to less selection pressure than vaccines, antivirals may lose efficacy over time as well and so will need to be continually reevaluated as a strategy for pandemic control.

In the aftermath of mass vaccination or infection, it is common to observe a transient phase of low incidence that gives way to recurrent oscillatory dynamics.[26,27] During this period, it is more challenging to measure vaccine efficacy directly, as establishing this requires a prevalent disease.[28] It is in situations like this that computer simulation studies, informed by correlates of protection, can be particularly useful.[29] A key result of our work is that while vaccine effectiveness declines in the wake of a large outbreak, this must be understood as a temporary effect, a corollary of the transient period in which population immunity levels are high. In the absence of ongoing vaccination, both waning immunity and vital dynamics will increase the fraction of the population susceptible to infection until there are enough for a resurgence.[30,31] Vaccines provide a safe mechanism for counteracting this and preventing or blunting the effect of future resurgences.

Our work demonstrates that the population immunity acquired over the first 2 years of the pandemic significantly reduces the impact per dose of future vaccinations. In many settings, such as the South Africa-like one considered here, this immunity is primarily a consequence of what Farmer et al. termed structural violence[32] and has been earned at great loss of life and health. While current and next-generation vaccines to fight future variants remain an essential part of any effective and equitable strategy, vaccine-based strategies alone are not sufficient. A layered

approach to respiratory disease transmission and the social conditions that exacerbate it is required to gain control of this pandemic and prevent the next.

Limitations of the study

Our modeling makes many assumptions that may limit the generalizability of our findings. Our analysis is based on a population similar to South Africa's in November 2021, but we do not capture many of the intricacies of the South African population (e.g., transmission dynamics, co-morbidities). Our analysis does not explicitly characterize co-morbidities or other factors such as immunosuppression at the individual level. For these populations who are at highest risk of severe outcomes if infected with SARS-CoV-2, ongoing boosters remain a highly relevant and valuable strategy. This study did not consider the use of antiviral pills that can be taken orally outside of a health-care setting, such as molnupiravir and Paxlovid. We are also not capturing new birth cohorts into the model with near complete susceptibility and no prior immunity to COVID beyond mother-to-child immune transference, for whom any vaccination strategy would be better than risking infection.[33,34]

While our model has a robust mechanistic representation of immune dynamics, we do not capture the process of affinity maturation that antibodies go through over time and after repeated exposures to an antigen that increase the breadth of the immune response even in the face of waning.[35] As a result, we may at times be underestimating the protection retained over time against infection. Additionally, we are not explicitly capturing the impact of original antigenic sin and its interaction with enhanced immunologic breadth following vaccination.[36]

STAR★METHODS

Detailed methods are provided in the online version of this paper and include the following:

- KEY RESOURCES TABLE
- RESOURCE AVAILABILITY
  - Lead contact
  - Materials availability
  - Data and code availability
- EXPERIMENTAL MODEL AND SUBJECT DETAILS
- METHOD DETAILS
  - Intra-host immunity model overview
  - Quantifying neutralizing antibodies as a correlate of protection
  - Covasim overview
  - Vaccine effectiveness over time
  - The effect of new variants
  - Trade-off between primary series and booster dose coverage
  - Variant-chasing and next generation vaccines
- QUANTIFICATION AND STATISTICAL ANALYSIS

SUPPLEMENTAL INFORMATION

Supplemental information can be found online at https://doi.org/10.1016/j.celrep.2023.112308.

ACKNOWLEDGMENTS

The findings, conclusions, and views expressed in this study are those of the author(s) and do not necessarily represent those of the Bill & Melinda Gates Foundation.

AUTHOR CONTRIBUTIONS

J.A.C., D.J.K., and M.F. conceived of the study. The Covasim model was developed and adapted for the analyses by J.A.C., C.C.K., R.M.S., R.G.A., and D.J.K. The intra-host immune model was developed by J.A.C., M.F., and R.M.S. J.A.C. programmed the analyses, produced visualizations, and drafted the manuscript. All authors reviewed and edited the manuscript. Supervision was provided by D.J.K. and M.F.

Received: March 10, 2022
Revised: September 22, 2022
Accepted: March 9, 2023
Published: March 15, 2023

REFERENCES

1. Baden, L.R., El Sahly, H.M., Essink, B., Kotloff, K., Frey, S., Novak, R., Diemert, D., Spector, S.A., Rouphael, N., Creech, C.B., et al. (2021). Efficacy and safety of the mRNA-1273 SARS-CoV-2 vaccine. N. Engl. J. Med. *384*, 403–416. https://doi.org/10.1056/NEJMoa2035389.
2. Shinde, V., Bhikha, S., Hoosain, Z., Archary, M., Bhorat, Q., Fairlie, L., Lalloo, U., Masilela, M.S.L., Moodley, D., Hanley, S., et al. (2021). Efficacy of NVX-CoV2373 covid-19 vaccine against the B.1.351 variant. N. Engl. J. Med. *384*, 1899–1909. https://doi.org/10.1056/NEJMoa2103055.
3. Logunov, D.Y., Dolzhikova, I.V., Shcheblyakov, D.V., Tukhvatulin, A.I., Zubkova, O.V., Dzharullaeva, A.S., Kovyrshina, A.V., Lubenets, N.L., Grousova, D.M., Erokhova, A.S., et al. (2021). Safety and efficacy of an rAd26 and rAd5 vector-based heterologous prime-boost COVID-19 vaccine: an interim analysis of a randomised controlled phase 3 trial in Russia. Lancet *397*, 671–681. https://doi.org/10.1016/S0140-6736(21)00234-8.
4. Sadoff, J., Le Gars, M., Shukarev, G., Heerwegh, D., Truyers, C., de Groot, A.M., Stoop, J., Tete, S., Van Damme, W., Leroux-Roels, I., et al. (2021). Interim results of a phase 1–2a trial of Ad26.COV2.S covid-19 vaccine. N. Engl. J. Med. *384*, 1824–1835. https://doi.org/10.1056/NEJMoa2034201.
5. Dorabawila, V., Hoefer, D., Bauer, U.E., Bassett, M.T., Lutterloh, E., and Rosenberg, E.S. (2022). Effectiveness of the BNT162b2 vaccine among children 5-11 and 12-17 years in New York after the emergence of the Omicron variant. Preprint at medRxiv. https://doi.org/10.1101/2022.02.25.22271454.
6. Sadarangani, M., Marchant, A., and Kollmann, T.R. (2021). Immunological mechanisms of vaccine-induced protection against COVID-19 in humans. Nat. Rev. Immunol. *21*, 475–484. https://doi.org/10.1038/s41577-021-00578-z.
7. Levin, E.G., Lustig, Y., Cohen, C., Fluss, R., Indenbaum, V., Amit, S., Doolman, R., Asraf, K., Mendelson, E., Ziv, A., et al. (2021). Waning immune humoral response to BNT162b2 covid-19 vaccine over 6 months. N. Engl. J. Med. *385*, e84. https://doi.org/10.1056/NEJMoa2114583.
8. Moss, P. (2022). The T cell immune response against SARS-CoV-2. Nat. Immunol. *23*, 186–193. https://doi.org/10.1038/s41590-021-01122-w.
9. Belizário, J.E. (2021). Immunity, virus evolution, and effectiveness of SARS-CoV-2 vaccines. Braz. J. Med. Biol. Res. *54*, e10725. https://doi.org/10.1590/1414-431X202010725.
10. Tarke, A., Sidney, J., Methot, N., Yu, E.D., Zhang, Y., Dan, J.M., Goodwin, B., Rubiro, P., Sutherland, A., Wang, E., et al. (2021). Impact of SARS-CoV-2 variants on the total CD4+ and CD8+ T cell reactivity in infected or vaccinated individuals. Cell Rep. Med. *2*, 100355. https://doi.org/10.1016/j.xcrm.2021.100355.
11. Bertoletti, A., Tan, A.T., and Le Bert, N. (2021). The T-cell response to SARS-CoV-2: kinetic and quantitative aspects and the case for their

protective role. Oxf. Open Immunol. *2*, iqab006. https://doi.org/10.1093/oxfimm/iqab006.

12. Nguyen, T.H.O., Cohen, C.A., Rowntree, L.C., Bull, M.B., Hachim, A., Kedzierska, K., and Valkenburg, S.A. (2021). T cells targeting SARS-CoV-2: by infection, vaccination, and against future variants. Front. Med. *8*.

13. WHO (2022). One Year since the Emergence of COVID-19 Virus Variant Omicron. https://www.who. int/news-room/feature-stories/detail/one-year-since-the-emergence-of-omicron.

14. Scheaffer, S.M., Lee, D., Whitener, B., Ying, B., Wu, K., Liang, C.-Y., Jani, H., Martin, P., Amato, N.J., Avena, L.E., et al. (2023). Bivalent SARS-CoV-2 mRNA vaccines increase breadth of neutralization and protect against the BA.5 Omicron variant in mice. Nat. Med. *29*, 247–257. https://doi.org/10.1038/s41591-022-02092-8.

15. Iwasaki, A. (2022). Opinion | the Answer to Stopping the Coronavirus May Be up Your Nose (The New York Times). https://www.nytimes.com/2022/05/16/opinion/covid-nasal-vaccine.html.

16. Kerr, C.C., Stuart, R.M., Mistry, D., Abeysuriya, R.G., Rosenfeld, K., Hart, G.R., Núñez, R.C., Cohen, J.A., Selvaraj, P., Hagedorn, B., et al. (2021). Covasim: an agent-based model of COVID-19 dynamics and interventions. PLoS Comput. Biol. *17*, e1009149. https://doi.org/10.1371/journal.pcbi.1009149.

17. Toor, J., Echeverria-Londono, S., Li, X., Abbas, K., Carter, E.D., Clapham, H.E., Clark, A., de Villiers, M.J., Eilertson, K., Ferrari, M., et al. (2021). Lives saved with vaccination for 10 pathogens across 112 countries in a pre-COVID-19 world. Elife *10*, e67635. https://doi.org/10.7554/eLife.67635.

18. Viana, R., Moyo, S., Amoako, D.G., Tegally, H., Scheepers, C., Althaus, C.L., Anyaneji, U.J., Bester, P.A., Boni, M.F., Chand, M., et al. (2022). Rapid epidemic expansion of the SARS-CoV-2 Omicron variant in southern Africa. Nature *603*, 679–686. https://doi.org/10.1038/s41586-022-04411-y.

19. Grabowski, F., Kochańczyk, M., and Lipniacki, T. (2022). The spread of SARS-CoV-2 variant Omicron with a doubling time of 2.0–3.3 Days can Be explained by immune evasion. Viruses *14*, 294. https://doi.org/10.3390/v14020294.

20. CDC COVID-19 Response Team (2021). SARS-CoV-2 B.1.1.529 (Omicron) variant — United States, december 1–8, 2021. MMWR Morb. Mortal. Wkly. Rep. *70*, 1731–1734. https://doi.org/10.15585/mmwr.mm7050e1.

21. Sprenger, K.G., Louveau, J.E., Murugan, P.M., and Chakraborty, A.K. (2020). Optimizing immunization protocols to elicit broadly neutralizing antibodies. Proc. Natl. Acad. Sci. USA *117*, 20077–20087. https://doi.org/10.1073/pnas.1919329117.

22. Singh, J., Pandit, P., McArthur, A.G., Banerjee, A., and Mossman, K. (2021). Evolutionary trajectory of SARS-CoV-2 and emerging variants. Virol. J. *18*, 166. https://doi.org/10.1186/s12985-021-01633-w.

23. Singh, D., and Yi, S.V. (2021). On the origin and evolution of SARS-CoV-2. Exp. Mol. Med. *53*, 537–547. https://doi.org/10.1038/s12276-021-00604-z.

24. Rochman, N.D., Wolf, Y.I., Faure, G., Mutz, P., Zhang, F., and Koonin, E.V. (2021). Ongoing global and regional adaptive evolution of SARS-CoV-2. Proc. Natl. Acad. Sci. USA *118*, e2104241118. https://doi.org/10.1073/pnas.2104241118.

25. Wen, W., Chen, C., Tang, J., Wang, C., Zhou, M., Cheng, Y., Zhou, X., Wu, Q., Zhang, X., Feng, Z., et al. (2022). Efficacy and safety of three new oral antiviral treatment (molnupiravir, fluvoxamine and Paxlovid) for COVID-19:a meta-analysis. Ann. Med. *54*, 516–523. https://doi.org/10.1080/07853890.2022.2034936.

26. McLean, A.R., and Anderson, R.M. (1988). Measles in developing countries. Part II. The predicted impact of mass vaccination. Epidemiol. Infect. *100*, 419–442. https://doi.org/10.1017/s0950268800067170.

27. Andreasen, V., Lin, J., and Levin, S.A. (1997). The dynamics of cocirculating influenza strains conferring partial cross-immunity. J. Math. Biol. *35*, 825–842. https://doi.org/10.1007/s002850050079.

28. Nikolay, B., Ribeiro dos Santos, G., Lipsitch, M., Rahman, M., Luby, S.P., Salje, H., Gurley, E.S., and Cauchemez, S. (2021). Assessing the feasibility of Nipah vaccine efficacy trials based on previous outbreaks in Bangladesh. Vaccine *39*, 5600–5606. https://doi.org/10.1016/j.vaccine.2021.08.027.

29. Halloran, M.E., Auranen, K., Baird, S., Basta, N.E., Bellan, S.E., Brookmeyer, R., Cooper, B.S., DeGruttola, V., Hughes, J.P., Lessler, J., et al. (2017). Simulations for designing and interpreting intervention trials in infectious diseases. BMC Med. *15*, 223. https://doi.org/10.1186/s12916-017-0985-3.

30. Chen, R.T., Weierbach, R., Bisoffi, Z., Cutts, F., Rhodes, P., Ramaroson, S., Ntembagara, C., and Bizimana, F. (1994). A "post-honeymoon period" measles outbreak in Muyinga sector, Burundi. Int. J. Epidemiol. *23*, 185–193. https://doi.org/10.1093/ije/23.1.185.

31. Rentsen, T., Enkhtuya, B., Nymadawa, P., Kobune, F., Suzuki, K., Yoshida, H., and Hachiya, M. (2007). Measles outbreak after a post-honeymoon period in Mongolia, 2001. Jpn. J. Infect. Dis. *60*, 198–199.

32. Farmer, P.E., Nizeye, B., Stulac, S., and Keshavjee, S. (2006). Structural violence and clinical medicine. PLoS Med. *3*, e449. https://doi.org/10.1371/journal.pmed.0030449.

33. Narayanaswamy, V., Pentecost, B.T., Schoen, C.N., Alfandari, D., Schneider, S.S., Baker, R., and Arcaro, K.F. (2022). Neutralizing antibodies and cytokines in breast milk after coronavirus disease 2019 (COVID-19) mRNA vaccination. Obstet. Gynecol. *139*, 181–191. https://doi.org/10.1097/AOG.0000000000004661.

34. Halasa, N.B., Olson, S.M., Staat, M.A., Newhams, M.M., Price, A.M., Boom, J.A., Sahni, L.C., Cameron, M.A., Pannaraj, P.S., Bline, K.E., et al. (2022). Effectiveness of maternal vaccination with mRNA COVID-19 vaccine during pregnancy against COVID-19–associated hospitalization in infants aged 6 Months — 17 states, july 2021–january 2022. MMWR Morb. Mortal. Wkly. Rep. *71*, 264–270. https://doi.org/10.15585/mmwr.mm7107e3.

35. Tang, J., Grubbs, G., Lee, Y., Huang, C., Ravichandran, S., Forgacs, D., Golding, H., Ross, T.M., and Khurana, S. (2021). Antibody affinity maturation and cross-variant activity following SARS-CoV-2 mRNA vaccination: impact of prior exposure and sex. EBioMedicine *74*, 103748. https://doi.org/10.1016/j.ebiom.2021.103748.

36. Pillai, S. (2022). SARS-CoV-2 vaccination washes away original antigenic sin. Trends Immunol. *43*, 271–273. https://doi.org/10.1016/j.it.2022.02.009.

37. Khoury, D.S., Cromer, D., Reynaldi, A., Schlub, T.E., Wheatley, A.K., Juno, J.A., Subbarao, K., Kent, S.J., Triccas, J.A., and Davenport, M.P. (2021). Neutralizing antibody levels are highly predictive of immune protection from symptomatic SARS-CoV-2 infection. Nat. Med. *27*, 1205–1211. https://doi.org/10.1038/s41591-021-01377-8.

38. Cohen, J.A., Stuart, R.M., Rosenfeld, K., Lyons, H., White, M., Kerr, C.C., Klein, D.J., and Famulare, M. (2021). Quantifying the role of naturally- and vaccine-derived neutralizing antibodies as a correlate of protection against COVID-19 variants. Preprint at medRxiv. https://doi.org/10.1101/2021.05.31.21258018.

39. Cromer, D., Steain, M., Reynaldi, A., Schlub, T.E., Wheatley, A.K., Juno, J.A., Kent, S.J., Triccas, J.A., Khoury, D.S., and Davenport, M.P. (2022). Neutralising antibody titres as predictors of protection against SARS-CoV-2 variants and the impact of boosting: a meta-analysis. Lancet. Microbe *3*, e52–e61. https://doi.org/10.1016/S2666-5247(21)00267-6.

40. Earle, K.A., Ambrosino, D.M., Fiore-Gartland, A., Goldblatt, D., Gilbert, P.B., Siber, G.R., Dull, P., and Plotkin, S.A. (2021). Evidence for antibody as a protective correlate for COVID-19 vaccines. Vaccine *39*, 4423–4428. https://doi.org/10.1016/j.vaccine.2021.05.063.

41. Kristiansen, P.A., Page, M., Bernasconi, V., Mattiuzzo, G., Dull, P., Makar, K., Plotkin, S., and Knezevic, I. (2021). WHO International Standard for anti-SARS-CoV-2 immunoglobulin. Lancet *397*, 1347–1348. https://doi.org/10.1016/S0140-6736(21)00527-4.

42. Pelleau, S., Woudenberg, T., Rosado, J., Donnadieu, F., Garcia, L., Obadia, T., Gardais, S., Elgharbawy, Y., Velay, A., Gonzalez, M., et al. (2021). Kinetics of the severe acute respiratory syndrome coronavirus 2 antibody response and serological estimation of time since infection. J. Infect. Dis. *224*, 1489–1499. https://doi.org/10.1093/infdis/jiab375.

43. Abdool Karim, S.S., and de Oliveira, T. (2021). New SARS-CoV-2 variants — clinical, public health, and vaccine implications. N. Engl. J. Med. *384*, 1866–1868. https://doi.org/10.1056/NEJMc2100362.

44. Castillo-Olivares, J., Wells, D.A., Ferrari, M., Chan, A.C.Y., Smith, P., Nadesalingam, A., Paloniemi, M., Carnell, G.W., Ohlendorf, L., Cantoni, D., et al. (2021). Analysis of serological biomarkers of SARS-CoV-2 infection in convalescent samples from severe, moderate and mild COVID-19 cases. Front. Immunol. *12*, 748291.

45. Davies, N.G., Abbott, S., Barnard, R.C., Jarvis, C.I., Kucharski, A.J., Munday, J.D., Pearson, C.A.B., Russell, T.W., Tully, D.C., Washburne, A.D., et al. (2021). Estimated transmissibility and impact of SARS-CoV-2 lineage B.1.1.7 in England. Science *372*, eabg3055. https://doi.org/10.1126/science.abg3055.

46. Bager, P., Wohlfahrt, J., Fonager, J., Rasmussen, M., Albertsen, M., Michaelsen, T.Y., Møller, C.H., Ethelberg, S., Legarth, R., Button, M.S.F., et al. (2021). Risk of hospitalisation associated with infection with SARS-CoV-2 lineage B.1.1.7 in Denmark: an observational cohort study. Lancet Infect. Dis. *21*, 1507–1517. https://doi.org/10.1016/S1473-3099(21)00290-5.

47. Faria, N.R., Mellan, T.A., Whittaker, C., Claro, I.M., Candido, D.d.S., Mishra, S., Crispim, M.A.E., Sales, F.C.S., Hawryluk, I., McCrone, J.T., et al. (2021). Genomics and epidemiology of the P.1 SARS-CoV-2 lineage in Manaus, Brazil. Science *372*, 815–821. https://doi.org/10.1126/science.abh2644.

48. Funk, T., Pharris, A., Spiteri, G., Bundle, N., Melidou, A., Carr, M., Gonzalez, G., Garcia-Leon, A., Crispie, F., O'Connor, L., et al. (2021). Characteristics of SARS-CoV-2 variants of concern B.1.1.7, B.1.351 or P.1: data from seven EU/EEA countries, weeks 38/2020 to 10/2021. Euro Surveill *26*, 2100348. https://doi.org/10.2807/1560-7917.ES.2021.26.16.2100348.

49. Cele, S., Gazy, I., Jackson, L., Hwa, S.-H., Tegally, H., Lustig, G., Giandhari, J., Pillay, S., Wilkinson, E., Naidoo, Y., et al. (2021). Escape of SARS-CoV-2 501Y.V2 from neutralization by convalescent plasma. Nature *593*, 142–146. https://doi.org/10.1038/s41586-021-03471-w.

50. Tarke, A., Sidney, J., Methot, N., Zhang, Y., Dan, J.M., Goodwin, B., Rubiro, P., Sutherland, A., da Silva Antunes, R., Frazier, A., et al. (2021). Negligible impact of SARS-CoV-2 variants on CD4 + and CD8 + T cell reactivity in COVID-19 exposed donors and vaccinees. Preprint at bioRxiv. https://doi.org/10.1101/2021.02.27.433180.

51. McMahan, K., Yu, J., Mercado, N.B., Loos, C., Tostanoski, L.H., Chandrashekar, A., Liu, J., Peter, L., Atyeo, C., et al. (2021). Correlates of protection against SARS-CoV-2 in rhesus macaques. Nature *590*, 630–634. https://doi.org/10.1038/s41586-020-03041-6.

52. Miller, N.L., Clark, T., Raman, R., and Sasisekharan, R. (2021). An antigenic space framework for understanding antibody escape of SARS-CoV-2 variants. Viruses *13*. https://doi.org/10.3390/v13102009.

53. Wilks, S.H., Mühlemann, B., Shen, X., Türeli, S., LeGresley, E.B., Netzl, A., Caniza, M.A., Chacaltana-Huarcaya, J.N., Daniell, X., Datto, M.B., et al. (2022). Mapping SARS-CoV-2 antigenic relationships and serological responses. Preprint at bioRxiv. https://doi.org/10.1101/2022.01.28.477987.

CellPress
OPEN ACCESS

STAR★METHODS

KEY RESOURCES TABLE

REAGENT or RESOURCE	SOURCE	IDENTIFIER
Deposited data		
Immune protection and response	Table S3 (excel spreadsheet) includes all data used for estimating neutralizing antibodies as a correlate of protection.	N/A
Software and algorithms		
Original code	All original code is publicly available at the following repository link: https://github.com/amath-idm/post-omicron (https://doi.org/10.5281/zenodo.7693637).	N/A

RESOURCE AVAILABILITY

Lead contact

Further information and requests for resources and source data should be directed to and will be fulfilled by the lead contact, Jamie Cohen (jamie.cohen@gatesfoundation.org)

Materials availability

This study did not generate new unique reagents.

Data and code availability

All data is available in the paper's supplemental information. All original code is publicly available at the following repository link: https://github.com/amath-idm/post-omicron (Mendeley Data: https://doi.org/10.5281/zenodo.7693637). Any additional information required to reanalyze the data reported in this paper is available from the lead contact upon request.

EXPERIMENTAL MODEL AND SUBJECT DETAILS

No experiments were conducted in this study.

METHOD DETAILS

Our analyses relied on two interlinked models: an intra-host immunity model, which is integrated into Covasim, an agent-based COVID-19 model that we parameterized to represent a South Africa like setting. We use Covasim for all our analyses: to estimate vaccine effectiveness over March 2020 to January 2022 within a South Africa like setting; to assess the possible impact of new variants over 2022 on the epidemic landscape; to estimate the trade-offs between primary series and booster coverage over 2022; and to investigate the impact of a variant-chasing vaccine strategy.

Intra-host immunity model overview

We adapted the neutralizing antibody (NAb) growth and decay model proposed in Khoury et al.[37] and used it to simulate individual immunity profiles. When first infected or vaccinated, individuals draw an initial level of NAbs from a lognormal distribution, i.e. $\log^2(NAb) \sim N(0,2)$. We then assume that NAbs increase linearly over the subsequent 21 days, before starting to exponentially decay with a half-life of 50 days for the first 6 months and a half-life of 250 days thereafter. Agents acquire NAbs from each different source of immunity (i.e., each variant or vaccine), and then we combine these to calculate "effective NAbs" (Equation 1) to determine how much protection is afforded against infection (Equation 5), symptomatic (Equation 6) and severe disease (Equation 7), accounting for cross-immune protection.[38]

$$Effective\ NAbs_j = \sum_{i=0}^{n} NAbs_i * cross\ immunity_{i,j} \quad \text{(Equation 1)}$$

Quantifying neutralizing antibodies as a correlate of protection

Various studies have related neutralizing antibodies to vaccine efficacy, showing that neutralization level is highly predictive of immune protection from symptomatic COVID-19, and that despite decaying immunity, protection from severe disease should be largely retained.[37,39,40] To extend these studies, we need to additionally calculate immune protection from infection and disentangle the relationship between NAbs and protection against primary infection, symptomatic, and severe disease.

We model protective efficacy as a function of neutralizing antibodies (NAbs), informed by data from vaccine immunogenicity and efficacy trials as well as observational studies. Extending upon the methodology from Earle[40] and Khoury,[37] we estimate efficacy for primary SARS-CoV-2 infection, symptomatic COVID-19, and severe disease jointly and calculate conditional efficacies for symptom- and severity-blocking given infection, revealing a direct model of NAbs as a correlate of protection. We additionally consider differences between the neutralizing antibodies generated from vaccines and infection while accounting for antibody waning between immunogenicity and vaccine efficacy studies.

In order to map neutralizing antibody (NAb) level to protective efficacy, we extracted cohort estimates from vaccine immunogenicity and efficacy trials as well as data on reinfection. In the absence of standardized assays to measure NAbs, normalization against a convalescent serum standard has been suggested as a method for providing greater comparability between results from different assays.[41] In order to compare the immunogenicity data with the efficacy endpoints, we accounted for waning that may have occurred across the timescales reported. We re-normalize the average NAb for each of the cohorts using an adaptation of the antibody kinetics functional form described in Khoury et al.[37] fit to cohorts of hospitalized patients and healthcare workers followed-up for eleven months after COVID-19 symptom onset.[42]

In the NAb re-normalization procedure, we used a model of immune waning to account for any decay in antibodies that may have occured between the time of the antibody assay collection and vaccine efficacy endpoints. To do so, we assume waning follows a 2-part exponential decay and fit the half-life and duration parameters to cohorts of French and Irish hospitalized patients and healthcare workers followed for up to eleven months after COVID-19 symptom onset.[42] Relative to the waning model used by Khoury et al., our model suggests both shorter initial decay of NAbs followed by a steeper long-term decay rate.

We also adjusted the reported neutralization level in settings where variants of concern were circulating at the time of efficacy endpoints based upon reported neutralization in convalescent and vaccine sera. In order to compute titer shifts for efficacy against variants of concern, NAbs were randomly shifted by a normally-distributed scaling factor,

$$\textit{titer shift}_s \sim N(\textit{titer shift mean}_s, \textit{titer shift SD}_s) \quad \text{(Equation 2)}$$

with a mean and standard deviation based upon titer shifts reported in the literature,[43] see Table. The values in Table can also be compared to those published in the meta-analysis by Cromer et al.,[39] which reports decreases in neutralisation titre to the alpha (1.6-fold), beta (8.8-fold), gamma (3.5-fold), and delta (3.9-fold) variants compared to wild-type.

Variant neutralization with wild type and vaccine sera

Variant	Wild type	Pfizer	Moderna	AstraZeneca
Alpha (B.1.1.7)	1.8 (0.41)	1.8 (0.41)	1.8 (0.41)	–
Beta (B.1.351)	13.3 (2.17)	10.3 (2.9)	12.4 (2.85)	–
Gamma (P.1)	8.66 (1.05)	–	–	–
Delta (B.1.617.2)	–	2.19 (0.05)	–	4.01 (0.32)

Values refer to the fold of reduction in neutralization with each variant relative to wild-type infection or vaccine source; standard deviation reported in parentheses.

We modeled three types of immunity: protection against infection, symptomatic disease, and severe disease and fit separate functions for immunity derived from infection and vaccination, which can be supported by the role of nucleocapsid-specific antibodies which are missing from some vaccines and may mechanistically explain why infection NAbs are more effective against infection.[44] We jointly estimated the relationship between NAbs and protective efficacy against infection, symptoms, and severe disease with study-specific random effects. $VE_{symp|inf,r}$ and $VE_{sev|symp,r}$ are unobserved, and we model them through the marginal efficacy against symptomatic and severe disease (Equations 3 and 4).

$$VE_{symp,r} = 1 - (1 - VE_{inf,r})\left(1 - VE_{symp|inf,r}\right) VE_{sev,r} = 1 - \quad \text{(Equation 3)}$$

$$\left(1 - VE_{symp,r}\right)\left(1 - VE_{sev|symp,r}\right) \quad \text{(Equation 4)}$$

We split vaccine efficacy into conditional parts to match the stages of the infection process and assumed both the efficacy against infection and the conditional efficacy against symptoms and severe disease are logit-log. The α and β parameters capture the intercept and slope in each equation, respectively (see Equations 5, 6, 7). Vaccine efficacy against infection, VE_{inf}, is the first stage that modulates the probability of infection given exposure. For people who get infected, symptomaticity is modulated by the conditional

vaccine efficacy given breakthrough infection, $VE_{symp|}$inf, and similarly severity is modulated by the conditional vaccine efficacy given a breakthrough symptomatic infection $VE_{sev|}$symp.

$$logit(VE_{inf,r}) = \alpha\inf + \alpha\, natinfdiff * infection_r + \beta\inf log(NAb_r) + \gamma_{r,s} \quad \text{(Equation 5)}$$

$$logit\left(VE_{symp|inf,r}\right) = \alpha\, symp|\inf + \beta\, symp|\inf log(NAb_r) \quad \text{(Equation 6)}$$

$$logit\left(VE_{sev|symp,r}\right) = \alpha\, sev|symp + \beta\, sev|symp\, log(NAb_r) \quad \text{(Equation 7)}$$

where NAb_r represents the average level of neutralizing antibodies across participants in record r, "infection" is a dummy variable that is equal to 1 when record r is immunity from infection and equal to 0 when record r is immunity from vaccination, and $\gamma_{r,s}$ is the random effect from study s associated with record r (some studies have multiple associated records).

We assumed study random effects are normally distribution with a mean of 0 and a standard deviation that is Cauchy distributed with a flat prior. For studies which reported efficacy against variants of concern, as part of the re-normalizing computational procedure, NAbs were randomly shifted by a normally-distributed scaling factor with a mean and standard deviation based upon titer shifts reported in the literature.[43,45–49] That is, the results marginalize over uncertainty in the NAb titer shift.

We estimated a Bayesian posterior for parameters in Equations 5, 6, and 7 with a Hamiltonian Monte Carlo method fit in Stan.

We find that neutralizing antibodies are strongly correlated with protection against SARS-CoV-2, symptomatic COVID-19, and severe disease (see Figure S2). In order to provide a 50 percent or higher reduction in the risk of symptomatic COVID-19, a vaccine would need to induce a NAb level at least one-tenth of the average convalescent level, and a one-third NAb level would be required to reduce the risk of infection by 50 percent or higher. While natural infection provides greater protection than vaccination for the same level of NAbs, all of the vaccines considered in this analysis meet the 50 percent risk reduction threshold and do not have the morbidity and mortality costs associated with COVID-19 infection. However, as will be discussed below, variants of concern challenge the efficacy of vaccines by reducing the neutralization levels and associated protection.

Given the fitted marginal efficacies above, we inferred the conditional protection against symptomatic and severe disease for individuals with a breakthrough infection and with breakthrough symptomatic disease. We find that any history of immunity would provide some protection against symptomatic and severe COVID-19, with a floor of approximately 37 percent reduction in the risk of symptomatic COVID-19 and 50 percent reduction in risk of severe disease conditional on a breakthrough infection or disease respectively. From that level, a percentage increase in NAbs would result in 3.8 percent (0.12, 11.75) reduction in risk against symptomatic disease a 7.9 percent (0.22, 26.15) reduction in the risk of severe COVID-19, conditional on a breakthrough infection and breakthrough disease respectively. While NAbs are correlated with protection against COVID-19, there may be other immune mechanisms, such as T cell response, that provide protection against symptomatic and severe disease.[50] These results suggest that even as antibodies wane and become insufficient to protect against infection, some immunity to symptomatic and severe disease will remain. We report the mean and standard deviation of the model parameters in Table S2.

Our results show that while NAbs induced by natural infection or vaccination wane and individuals may lose sterilizing immune protection, immune memory is likely to be retained long-term to provide significant protection against severe disease, even in the face of immune-evading variants. This suggests that neutralizing titers play a large role in preventing infection, but that other immunologic factors may play a more dominant role in controlling infection once it occurs.

Our modeling approach relies on estimating a relationship between NAbs and protection against infection, symptomatic disease, and severe disease, and the data used to establish these estimates are scarce and uncertain, especially for low levels of NAbs. While a full individual-level model would be ideal, we relied upon published cohort averages and tried to account for variation and heterogeneity between studies using study-level random effects. We also assume that the antibody kinetics are identical for vaccine- and naturally-derived NAbs. We do not specifically model cellular immune responses, although they are likely to also influence disease symptomaticity and severity and to have different kinetic profiles than antibodies.[50,51]

Covasim overview

Covasim is an open-source agent-based model developed by the Institute for Disease Modeling with source code and documentation available at https://covasim.org. Covasim simulates individuals interacting via population networks over time, and tracks disease transmission and progression as well as the effects of interventions including symptomatic and asymptomatic testing, isolation, contact tracing, and quarantine, as well as other non-pharmaceutical interventions (NPIs) such as physical distancing, hygiene measures, and protective equipment such as masks. A comprehensive overview of the methodology underlying the model is provided in Kerr et al.[16]

We use Covasim's default network generation algorithm to create four contact networks, representing households, schools, workplaces, and community settings. This algorithm is described in greater detail in Kerr et al.,[16] but in brief, it creates households with $h \sim Poisson(3)$ members, assigns children aged 6–22 to schools and adults aged 22–65 to workplaces with $s \sim Poisson(20)$ and $w \sim Poisson(16)$ daily contacts respectively, and gives each agent an additional $c \sim Poisson(20)$ contacts from others in the community.

Individuals who contract SARS-CoV-2 for the first time then progress through the stages of infection: exposed, infectious (asymptomatic, presymptomatic, mild, severe, or critical), before either recovering or dying, with the probabilities of disease progression dependent on age. Individuals are allowed to recover from any of the disease stages, but only critically ill individuals have a non-zero probability of dying. The model includes individual heterogeneity in infectiousness and in the time spent in each disease state.

Recovered individuals are assumed to be susceptible to reinfection, but their relative susceptibility is modified by a factor that reflects the degree of protective immunity afforded by their prior infection.

As well as belonging to these disease states, agents in the model also have individual attributes that govern their movement through the model over time, such as age, infection history, vaccination history, and whether they have been tested, diagnosed, or contact-traced.

Vaccine effectiveness over time

For these analyses, we initialized a population of 60,000 agents with the same age structure as South Africa. Each agent is assumed to represent 1000 individuals, i.e. a population of 60 million. We introduced wild-type, Beta, Delta, and Omicron infections into the population respectively on March 01, 2020, October 15, 2020, May 01, 2021, and October 25, 2021 with variant-specific characteristics as described in Table S2. In the three months following the introduction of each variant, we assume that NPIs were in place that reduced transmission by 40–70 percent relative to a baseline of no restrictions.

Vaccine efficacy is not a direct input into the model, but rather an output of our immune model. Population-level effectiveness varies over time, decreasing in the immediate wake of each new wave of infections and increasing thereafter. In order to estimate how vaccine effectiveness varied over March 2020 to April 2022, we simulate a vaccine trial. We randomly selected agents to vaccinate for the vaccine arm and participants with no vaccine as the placebo arm. We note that individuals with prior infection are not excluded from either arm of the study, meaning that both the vaccine and placebo arm will be tainted by changing levels of prior immunity over time. We then calculate vaccine efficacy and effectiveness against infection, symptomatic disease, and severe disease.

The effect of new variants

For these analyses, we again create a population of 60,000 agents, but this time we need to ensure that the simulated agents have immunity profiles corresponding to that of the South African population in February 2022. Rather than simulating South Africa's epidemic directly, we use an algorithm for imprinting immunity, which is included as part of the Covasim model. Essentially, this algorithm takes the overall duration and peak height of each wave and uses this to determine how many people to infect and at what point in time. This dispenses with the need to track infections over contact networks, and is considerably faster than simulating transmission over a 2 year period, although it does mean that we lose some of the nuance of infection pockets occurring within particular socio-geographic groupings. We also imprint a history of vaccination within the population, assuming that 50 percent of the agent population had been vaccinated by February 2022 with two doses of a vaccine with characteristics assumed based on Pfizer vaccines in these observed trials. After imprinting immunity from the wild-type, Beta, Delta, and Omicron waves on our agent population, as well as the history of vaccination, each agent within the model has a level of effective NAbs (as per Equation 1) in February 2022.

Into this population of agents, we introduce one of three hypothetical variants that vary in terms of their antigenic distance from existing sources of immunity[52,53] and their virulence (see Table). We note that these characteristics are not intended to represent a comprehensive range of the likely qualities of newly emerging variants, but rather represent a small and illustrative subsection of outcomes, designed to highlight the general principle of how vaccine strategies are affected by variant properties. We chose 3.5-fold more virulent than wild-type as an upper bound of virulence based upon the estimated virulence of the Delta variant. We chose fold reductions of 2 and 50 to represent upper and lower bounds on expected antigenic drift of a new variant. In the absence of a seasonal or known pattern of variant emergence, we randomly introduce the new variant on three dates (2, 4, and 8 months after the introduction of the last variant).

New variant characteristics

Variant	Severity	Omicron fold reduction	WT fold reduction
Emerged from Omicron	1	2	50
Emerged from WT, more virulent (Delta-like)	3.5	50	2
New antigenic cluster, more virulent	3.5	50	50

Severity values refer to the change compared with wild type, and fold reduction refers to change in neutralization with each variant relative to Omicron or all other (wild-type cluster, including prior variants and vaccines). All new variants were assumed to be 3.5× as transmissible per contact compared with wild type. WT, wild type.

Trade-off between primary series and booster dose coverage

We begin with the population of agents with immune history. We then sweep over a combination of primary series and booster series coverage levels, from 0 to 100 percent of the eligible population. Eligibility is defined as people over 12 years of age (matching expanded age of vaccination) and unvaccinated for the primary series and over 12 years of age and vaccinated at least 6 months

prior for the booster dose. Both the primary and booster-dose series take three months to achieve the target coverage level. We quantify cumulative infections, severe cases, and deaths averted compared to no vaccination for the next 10 months and assuming no further increase in vaccination over this period.

Variant-chasing and next generation vaccines

Once again, we begin with the population of agents with immune history. To evaluate the impact of a variant-chasing vaccine strategy, we consider a variant that emerges further antigenically from all prior immunity, meaning it escapes 98 percent of all prior sources of immunity. A perfectly matched vaccine is rolled out on a set of dates following introduction of the variant. We assume it takes 30 days to reach the target coverage from the date of vaccine roll-out and that the variant-specific vaccine requires two doses to achieve the target efficacy, which we define as the same level of neutralizing antibody as the two-dose primary vaccine series.

In parallel, we explore next generation vaccines in the model by comparing the impact of vaccines with combinations of these properties delivered today against an emergent immune evading variant 8 months following Omicron. We specifically define a more broadly neutralizing vaccine as having complete cross-immunity against future variants and a more durable vaccine as waning at a significantly slower rate than current vaccines.

QUANTIFICATION AND STATISTICAL ANALYSIS

Statistical analysis for the correlate of protection work was performed using a Hamiltonian Monte Carlo method in Stan.

Cell Reports, Volume *42*

Supplemental information

The changing health impact of vaccines

in the COVID-19 pandemic: A modeling study

Jamie A. Cohen, Robyn M. Stuart, Jasmina Panovska-Griffiths, Edinah Mudimu, Romesh G. Abeysuriya, Cliff C. Kerr, Michael Famulare, and Daniel J. Klein

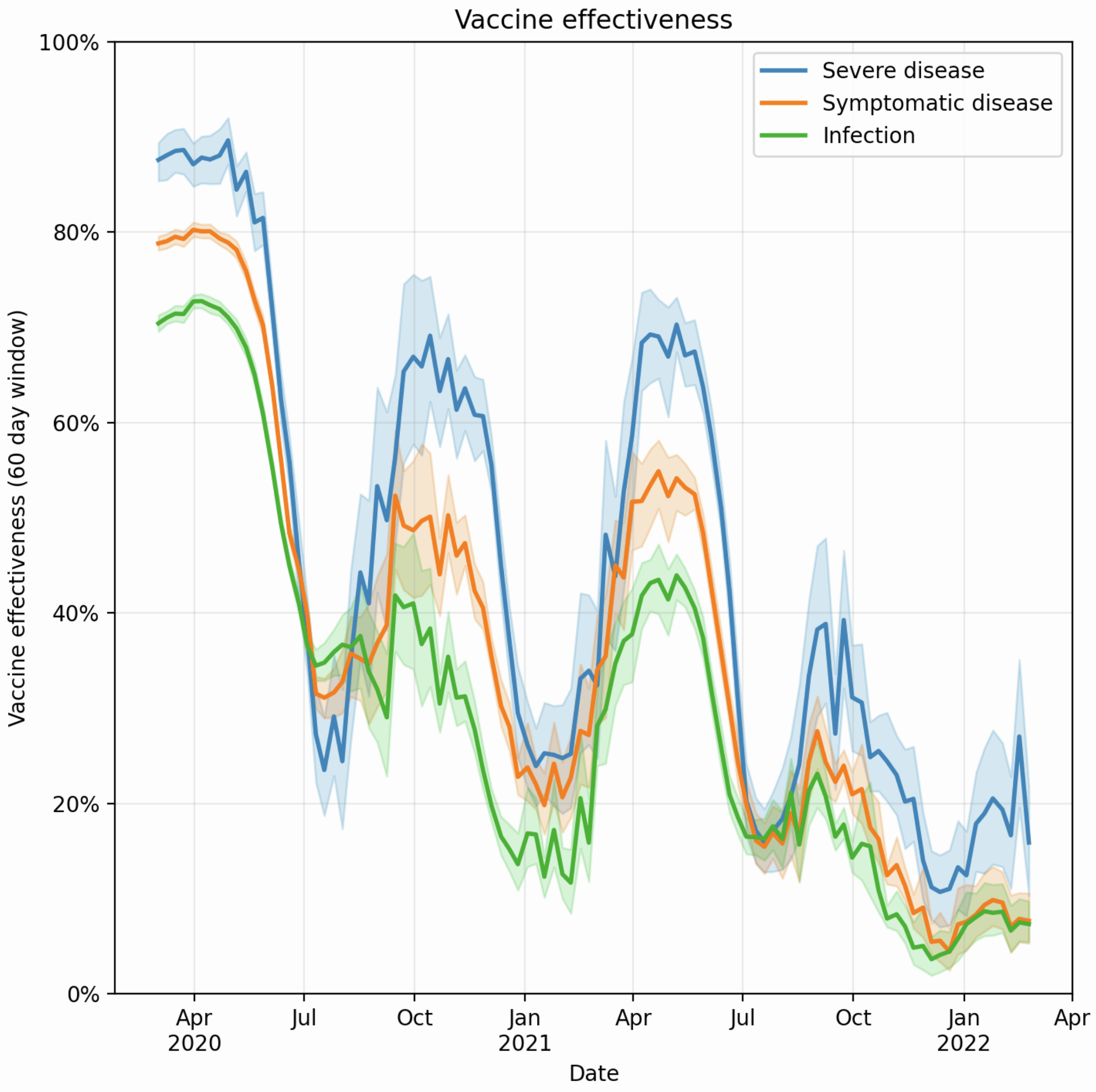

Fig. S1. Vaccine effectiveness against infection, symptomatic COVID and severe disease.

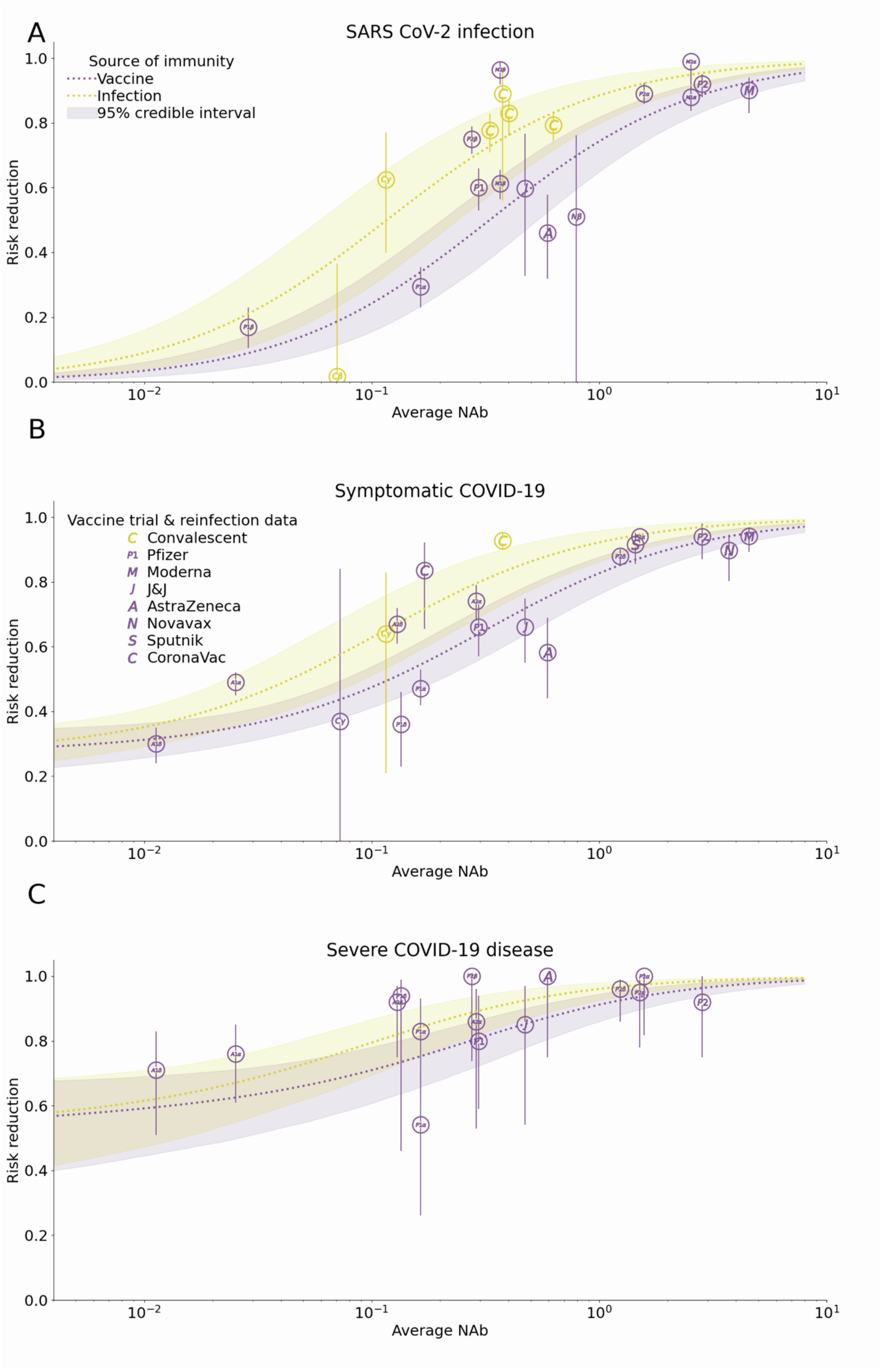
A
SARS CoV-2 infection
Source of immunity
Vaccine
Infection
95% credible interval
Risk reduction
Average NAb
B
Symptomatic COVID-19
Vaccine trial & reinfection data
C Convalescent
P1 Pfizer
M Moderna
J J&J
A AstraZeneca
N Novavax
S Sputnik
C CoronaVac
Risk reduction
Average NAb
C
Severe COVID-19 disease
Risk reduction
Average NAb

Fig. S2. Conditional protection against symptomatic and severe disease given a breakthrough infection and breakthrough symptoms, respectively.

	Mean	Standard Deviation
αinf	1.08	0.226
αnatinfdiff	1.0	0.403
βinf	0.967	0.055
αsymplinf	-0.739	0.187
βsymplinf	0.038	0.0317
αsevlsymp	-<br>0.0143	0.311
β sevlsymp	0.0799	0.078
α_γ	0.639	0.177

Table S1. Fitted parameter values based upon HMC algorithm with 30,000 iterations across 5 chains.

Variant	Infectivity	Severity	WT cross-immunity	Beta cross-immunity	Delta cross-immunity	Pfizer cross-immunity
Beta	1	3.6	6.6%	100%	8.6%	10%
Delta	2.2	3.2	37.4%	8.6%	100%	33%
Omicron	3.0	0.8	5%	4%	4%	2.5%

Table S2. Variant characteristics. Infectivity and severity values refer to the change in per-contact transmission rate and per-infection probability of developing severe disease compared to wild-type. Cross-immunity refers to how much prior neutralizing antibodies are retained to effectively protect against each variant.
